# Reducing missed appointments in general practice: evaluation of a quality improvement programme in East London

**DOI:** 10.3399/bjgp20X713909

**Published:** 2020-12-01

**Authors:** Tom Margham, Crystal Williams, Jack Steadman, Sally Hull

**Affiliations:** Jubilee Street Practice, London.; Institute of Population Health Sciences, Queen Mary University of London, London.; NHS Tower Hamlets Clinical Commissioning Group, London.; Institute of Population Health Sciences, Queen Mary University of London, London.

**Keywords:** general practice, non-attendance, quality improvement

## Abstract

**Background:**

Missed appointments are common in primary care, contributing to reduced clinical capacity. NHS England has estimated that there are 7.2 million missed general practice appointments annually, at a cost of £216 million. Reducing these numbers is important for an efficient primary care sector.

**Aim:**

To evaluate the impact of a system-wide quality improvement (QI) programme on the rates of missed GP appointments, and to identify effective practice interventions.

**Design and setting:**

Practices within a clinical commissioning group (CCG) in East London, with an ethnically diverse and socially deprived population.

**Method:**

Study practices engaged in a generic QI programme, which included sharing data on appointment systems and Did Not Attend (DNA) rates. Fourteen out of 25 practices implemented DNA reduction projects, supported by practice-based coaching. Appointment data were collected from practice electronic health records. Evaluation included comparisons of DNA rates pre- and post-intervention using interrupted times series analysis.

**Results:**

In total, 25 out of 32 practices engaged with the programme. The mean DNA rate at baseline was 7% (range 2–12%); 2 years later the generic intervention DNA rates were 5.2%. This equates to a reduction of 4030 missed appointments. The most effective practice intervention was to reduce the forward booking time to 1 day. The practice that made this change reduced its mean DNA rate from 7.8% to 3.9%.

**Conclusion:**

Forward booking time in days is the best predictor of practice DNA rates. Sharing appointment data produced a significant reduction in missed appointments, and behaviour change interventions with patients had a modest additional impact; in contrast, introducing structural change to the appointment system effectively reduced DNA rates. To reduce non-attendance, it appears that the appointment system needs to change, not the patient.

## INTRODUCTION

Non-attendance for appointments is a problem that is widely experienced across healthcare settings. In primary care, Did Not Attends (DNAs) result in wasted appointments, reduced clinical capacity, and inequality of access to health care.^1^ NHS England reports that ‘missed GP appointments cost millions’ *,* calculating that 5% — more than 15 million — of appointments in primary care are missed every year, of which 7.2 million are booked GP appointments. ^2^ This equates to 1.2 million GP hours, with estimated NHS costs of £216 million annually. To address these costs, NHS England exhorts patients to *‘cancel appointments, rather than just not show up’*.^2^ Such reports by NHS England also generate media headlines: *‘GP appointments missed by 20 000 patients each day. Failure to attend wastes £200 million a year’*.^3^ This comes at a time of constrained NHS finances and lengthening waiting times to see a GP.^4^

Non-attendance as a problem is a relatively recent phenomenon, arising from the creation of appointment systems. Between 1951 and 1981, the proportion of practices in the UK using an appointment system increased from 2% to 88%.^5^ GPs and reception teams typically cite patient factors as the main driver for non-attendance, and judge patients who do not attend to be forgetful, leading chaotic lives, or not valuing the appointment enough to attend.^6^

Reception teams feel the impact of DNAs on capacity most acutely, as they try to fit patients in to scarce appointments.^7^ Many GPs, however, might challenge the assertion that all DNAs represent ‘waste’ — the time is filled with other work, particularly when the DNA occurs late in a surgery session.^7^ In addition, DNAs can be an indicator of patient risk — for example, they could serve as a pointer to possible neglect of a child repeatedly not brought to appointments. There are also vulnerable patients, for whom a missed appointment may trigger a proactive check on welfare.^1^ Patients report competing demands that influence their attendance at the surgery: fitting appointments around work and family commitments, difficulty getting an appointment, and long wait times are reported as factors influencing non-attendance.^7^^,^^8^ Further, busy telephone lines have been reported to act as a barrier to cancelling appointments.^7^^,^^8^

Viewed from a systems perspective, the percentage of DNAs is a useful indicator of the ‘health’ of an appointment system. In one study, clinical commissioning group (CCG) practices with good access (based on national surveys and Healthwatch data) were shown to have lower DNA rates.^9^ However, there are conflicting motivations for addressing the problem of DNAs. For many GPs, DNAs represent a chance to catch up or take a comfort break during a long surgery session. In UK general practice, with capitation as the largest funding element, there is no direct financial incentive to address non-attendance. This contrasts with healthcare systems based on item-of-service or attendance payments. However, addressing DNA rates is important if the primary care sector is to be efficient. A missed appointment does not necessarily mean the patient’s reason for consulting has resolved: the patient may still present, but at less convenient times and in less appropriate settings, with the additional health and financial implications that frequently accompany a worsening condition.^10^

**Table:** How this fits in

Missed appointments (Did Not Attends [DNAs]) in general practice reduce clinical capacity and waste money. Most research on reducing DNAs focuses on changing patient behaviour to optimise the existing appointment system. This study shows the impact of quality improvement coaching, including sharing appointment system data, among practices in one clinical commissioning group. A case study illustrates how structural change to the practice’s appointment system produced sustained reductions in DNA rates.

When considering DNA rates, it is important to recognise that GP surgeries, essentially, run two systems in parallel:

- reactive care, which comprises most GP workload; and
- planned or proactive care for long-term conditions, which is provided by the nursing and healthcare assistant workforce.

These two systems function differently and should be considered separately. In East London, a consistent finding is that DNA rates for nursing or community pharmacist appointments (proactive care) are twice that of GP appointments (reactive care). As GP appointments comprise the larger volume and cost to the service, they are the focus of this study. This study aimed to:

- evaluate the impact on practice DNA rates of a system-wide quality improvement (QI) programme, which includes data sharing on appointment systems and DNA rates; and
- compare the effectiveness of different interventions at reducing DNA rates for GP appointments.

## METHOD

### Setting

This QI project took place in East London primary care between April 2016 and March 2019. All 32 practice teams in NHS Tower Hamlets Clinical Commissioning Group (CCG) were invited by the project organisers to participate; 25 out of 32 practices, with a registered population of 238 090, engaged with the project. In the 2011 UK Census, it is recorded that almost half of the population in this CCG is of non-white ethnic origin,^11^ and the locality falls in the lowest decile for social deprivation in England.^12^

All practices in the CCG use the EMIS Web clinical system and have access to Edenbridge Apex, a business intelligence and data visualisation platform with an Application Programming Interface with EMIS Web. In-practice configuration of Edenbridge Apex ensured that the software reliably captured GP appointment activity.

**Figure 1.**
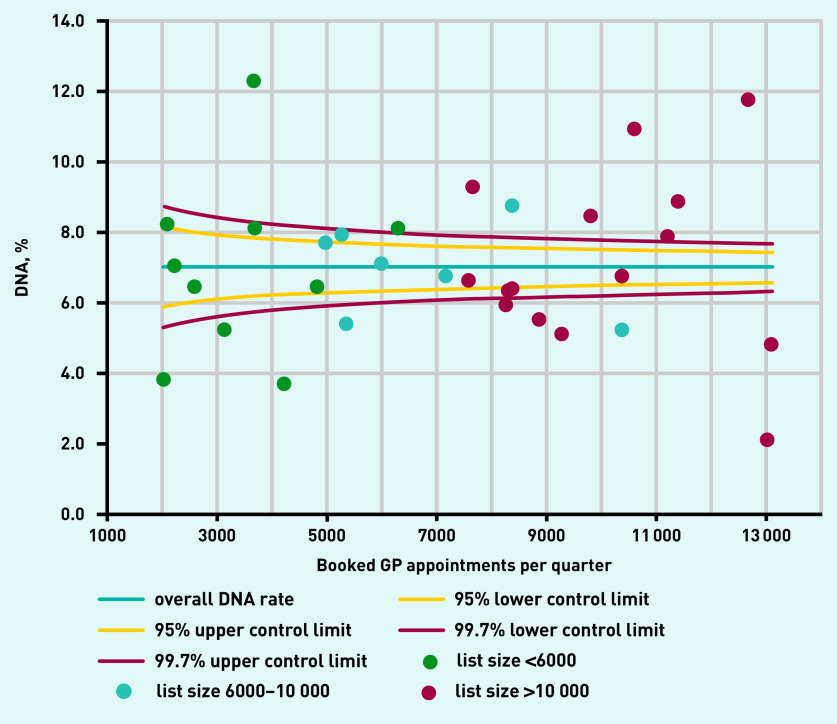
**Pre-intervention quarterly practice DNA rates plotted against number of booked appointments, January–March 2016.** **DNA = Did Not Attend.**

### Intervention

Enabling Quality Improvement in Practice (EQUIP) is a QI programme funded by Tower Hamlets CCG. The aim is to empower practice staff to make, and evaluate, operational changes that have a tangible impact on staff and patient satisfaction (https://equiptowerhamlets.nhs.uk/). Practices taking part in the EQUIP programme (‘EQUIP practices’):

- sign a data-sharing agreement;
- have access to QI training — this includes a half-day basic training session and coached learning sets with 3 days of face-to-face learning over 4 months;
- attend a facilitated ‘data wall’ session, in which practice teams have a 360-degree view of their practice using the 5Ps — purpose, process, patients, people, patterns — framework.^13^ This is a tested method encouraging team members to ask new questions about their system. Discoveries made using the 5Ps help teams select their own themes for improvement. Data walls contain detailed information on patterns of access, time lost to DNAs, and DNA rates plotted against the forward booking time;
- regular in-practice coaching from external improvement coaches who support the projects each practice chooses to undertake; and
- access to LifeQI, an online project management platform, allowing teams to track their improvement work (https://www.lifeqisystem.com/).

Practice-generated improvement themes included managing test results, increasing use of online services, and improving document workflow. During the study period, 14 out of 25 practices tested approaches to reduce DNA rates. Most practices chose patient behavioural interventions, such as publicising the number of appointments lost to DNAs, SMS reminders, hotline/text cancellation services, or telephone reminders for those with a history of DNAs. Collectively, these were characterised as ‘nudge’ changes, as they focus on patient behaviour change and encouraging altruistic behaviour to enable the existing system to run effectively. One practice instituted a systematic change to the appointment system by reducing the maximum booking time from 1 month to 1 working day.

### Data sources

Monthly appointment data for practices were collected from EMIS Web between April 2014 and March 2019. Data included the number and type of appointments booked, DNAs, and length of time between booking and appointment. For each practice that undertook a project aimed at reducing DNAs, the intervention start date was recorded. The monthly practice DNA rate was calculated as the number of DNAs divided by the number of appointments booked. The DNA status of an appointment is automatically recorded on EMIS Web at 10 minutes after the booked time; as this is an automatic setting within EMIS, the data were not susceptible to practice variation in data collection.

Patient-level data were pseudonymised at source and extracted from individual practices for analysis using Edenbridge Apex software.

### Data analysis

In order to investigate whether the DNA rate declined after a generic intervention in the practices participating in the EQUIP programme, or following specific DNA project work in the other practices, interrupted time series analysis, based on Poisson regression models, was used.^14^ The main outcome was a difference in slope of the DNA trend line pre- and post-intervention. As practices had different intervention start dates, these were taken into account during analysis. All models were corrected for over-dispersion. Analysis was undertaken in Stata (version 16.0).

## RESULTS

Data from all 32 practices in Tower Hamlets CCG were available for analysis, comprising >4 million booked appointments between April 2014 and March 2019. Before the project start (in April 2016 the mean DNA rate across all practices was 7.0%, with a range of 2.1–12.2% (Figure 1). This variation in DNA rates between practices was unrelated to practice size: smaller practices with a list size ≤6000 showed a similar range of variation as practices with a list size ≥10 000, as the baseline data in Table 1 show.

**Table 1.** Baseline rate of missed appointments for EQUIP practices (*n* = 25) and non-EQUIP (*n* = 7) practices in East London, April–June 2016

	**List size**	**Booked appointments, *n***	**DNAs, *n***	**DNA rate per 100 patients**	**DNA rate per 100 appointments**
**EQUIP practices**					
1	1935	2031	77	4.0	3.8
2	3416	2620	169	4.9	6.5
3	4408	3116	162	3.7	5.2
4	4769	4243	156	3.3	3.7
5	4904	3706	453	9.2	12.2
6	5215	4852	311	6.0	6.4
7	6554	5019	383	5.8	7.6
8	6616	10 413	541	8.2	5.2
9	7300	5384	287	3.9	5.3
10	7691	5296	416	5.4	7.9
11	8312	8411	730	8.8	8.7
12	9020	6038	426	4.7	7.1
13	9647	7213	483	5.0	6.7
14	10 081	8894	486	4.8	5.5
15	10 523	7626	501	4.8	6.6
16	10 954	11 246	879	8.0	7.8
17	11 017	9305	473	4.3	5.1
18	11 259	13 082	270	2.4	2.1
19	11 416	7696	710	6.2	9.2
20	11 648	8428	536	4.6	6.4
21	11 756	8357	525	4.5	6.3
22	12 897	10 419	699	5.4	6.7
23	14 080	10 663	1159	8.2	10.9
24	19 231	12 726	1486	7.7	11.7
25	23 442	13 131	623	2.7	4.7
Total	238 091	189 915	12 941	5.4	6.8

**Non-EQUIP practices**					
26	3621	2103	172	4.8	8.2
27	2926	2236	157	5.4	7.0
28	4940	3734	301	6.1	8.1
29	5942	6324	510	8.6	8.1
30	10 491	8291	488	4.7	5.9
31	13 036	9840	829	6.4	8.4
32	12 016	11 449	1012	8.4	8.8
Total	52 972	43 977	3469	6.5	7.9

DNA = Did Not Attend. EQUIP = Enabling Quality Improvement in Practice, the quality-improvement programme.

**Figure 2.**
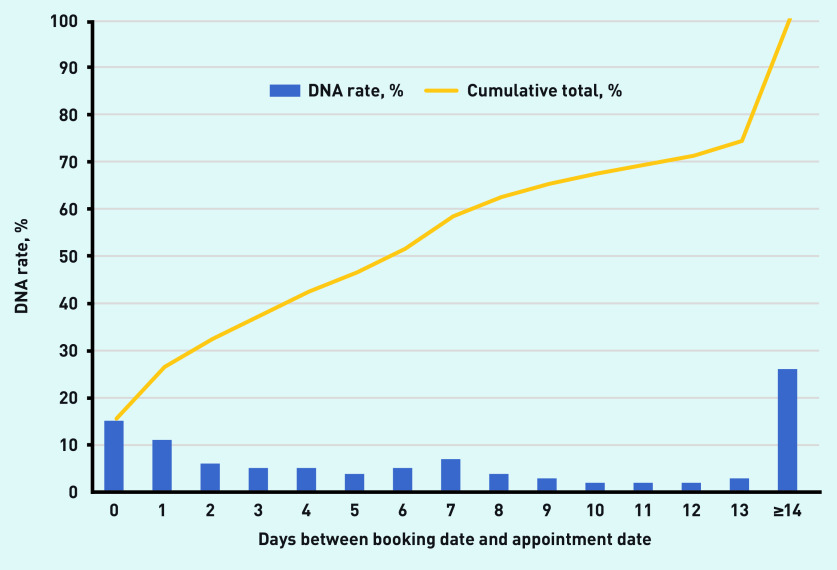
**Singular and cumulative DNA rates plotted against appointment delay in days.^a^** ^a^**Based on >4 million appointments from all 32 practices, April 2014–March 2019. DNA = Did Not Attend.**

Baseline data for all practices in the CCG showed a positive association between the DNA rate and the length of time, in days, between booking the appointment and the appointment date (Figure 2). Booking in advance beyond 2 days accounted for 75% of the DNA total.

**Figure 3.**
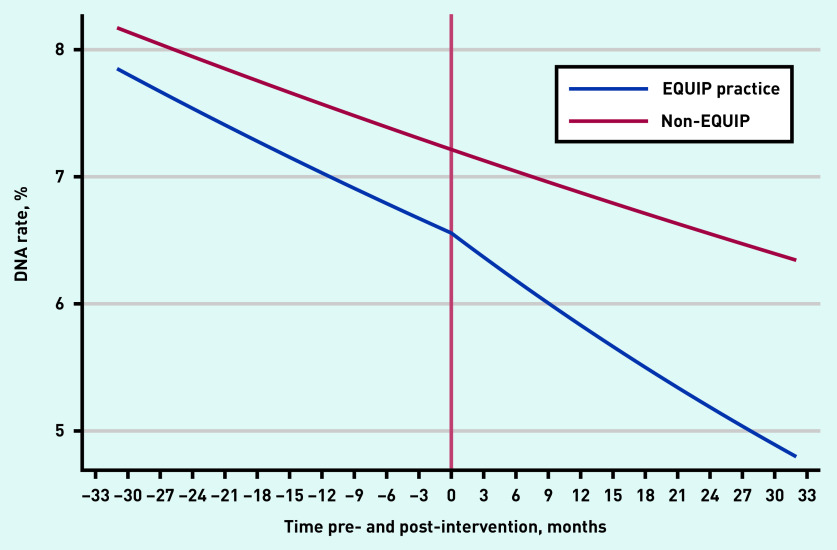
**Quarterly trends in percentage of DNAs: comparison of EQUIP practices (n = 25), with control practices (n = 7), using interrupted times series analysis, April 2014–March 2019.^a^** ^a^**Adjusted for the different intervention start date of each EQUIP practice. For the 25 EQUIP practices, the pre-intervention monthly change in DNA rates was 0.993 (95% CI = 0.992 to 0.994). The post-intervention monthly change in DNA rates was 0.990 (95% CI = 0.987 to 0.992); P = 0.001. For the seven non-EQUIP comparison practices, the monthly change in DNA rates was 0.996 (95% CI = 0.995 to 0.997). DNA = Did Not Attend. EQUIP = Enabling Quality Improvement in Practice, the quality improvement programme.**

To investigate whether the DNA rate declined after the generic intervention in the 25 EQUIP practices, an interrupted time series analysis was conducted; this compared the 25 EQUIP practices with the seven ‘non-EQUIP practices’, which acted as natural controls. For all 25 EQUIP practices, the observed DNA rate was 0.052 (5.2%) at 24 months after the intervention (Figure 3). Had the intervention not been in place, the predicted rate of DNA (extrapolated from the pre-intervention rates) would be 5.8%. This difference is equivalent to an absolute reduction of 4031 DNAs per year (based on 762 851 booked appointments in the 25 EQUIP practices 2018). As a DNA costs on average £30,^2^ this represents an estimated saving of £120 930 per year for all 25 EQUIP practices.

A similar analysis was used to examine the change in DNA rates following specific DNA reduction projects undertaken in 14 EQUIP practices. The 11 comparison EQUIP practices undertook a range of QI projects unconnected with DNA rates. This showed that practices engaging with DNA reduction projects had a modest additional drop in DNA rates compared with practices undertaking other projects, but the final DNA rates remained above the rates for the comparison practices (Supplementary Figure S1).

### Case study

Of the 14 practices that were working on DNA reduction projects, one implemented a systematic change to its appointment system. Practice X, with a registered population of 9000 patients, faced a shortfall of appointment capacity due to a reduction in GP numbers. Just prior to the intervention, it had a DNA rate of 9.5% for GP appointments — equivalent to >6 hours of GP time each week.

Using Edenbridge Apex software, the practice team identified that >70% of its DNAs occurred when the gap between the date of the booking and that of the appointment was >2 days; as such, it decided to test reducing the advance appointment booking period from 28 days to 1 working day. The team discussed which groups of patients might be disadvantaged by this approach — namely, carers and patients with specific advocacy needs that require advance booking — and exempted them from the policy. Following the intervention, DNAs rapidly fell to 3–4%; this level was maintained until the end of the study period in March 2019.

The change did result in some adverse effects: the supply of appointments was still insufficient to meet demand and patients had to call again if there were no appointments the next working day. As mitigation, the practice now has a small number of advance appointments and allows online booking a few days in advance.

**Figure 4.**
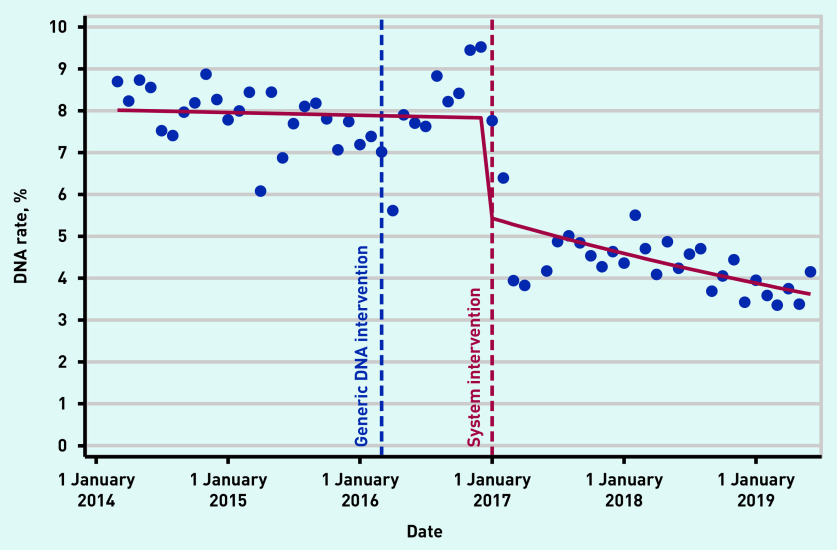
**Practice X case study: monthly percentage of DNAs pre- and post-systematic change intervention, using interrupted times series analysis.^a^** *^a^**A generic quality improvement intervention that commenced in April 2016 was followed by a systematic change to the practice’s appointment system in January 2017. Before the system intervention, the monthly change in DNA rates was 0.999 (95% CI = 0.996 to 1.003). Post-intervention, the monthly change in DNA rates was 0.986 (95% CI = 0.980 to 0.992).*** **P** ***= <0.001 for difference in slopes pre- and post-intervention. DNA = Did Not Attend.***

Prior to the system change, the average DNA rate for Practice X was 7.8%; after making the change, the DNA rate fell rapidly, reaching 3.9% at 24 months post-intervention (Figure 4). This case study demonstrates that addressing DNAs alone is insufficient; the issue needs to be seen in the broader context of practice work on capacity and demand.

## DISCUSSION

### Summary

Before the intervention, practice DNA rates ranged from 2% to 12% and showed a consistent relationship with the length of advance booking. To the authors’ knowledge, this is the first study to demonstrate the impact of data sharing and generic QI training on appointment systems, DNA rates, and demand management across practices in a local health economy and generic QI training on appointment systems and demand management across practices in a local health economy. The reduction in DNAs across the 25 study practices equated to 4031 gained appointments and a potential saving of £120 930 per year.

Most practices chose to test out patient behaviour change interventions, leaving their appointment system unchanged. The data presented here support previous findings that such interventions have only a modest impact. The greatest reduction in DNA rates was made by the practice that made a systematic change; further, the reduction was sustained to the end of the study period.

### Strengths and limitations

This study is based on data from >4 million GP appointments made over 5 years, in a multi-ethnic, urban area of deprivation, in which most practices had DNA rates above the national average (5%). The data on appointment booking and DNA rates are robust, being a core element of the computer system used by all practices in the study locality. Although practices started their DNA interventions at different times, it was possible to take account of this in the evaluation.

Study weaknesses include the heterogeneity of practice behavioural change interventions, and the fact that only one practice made a structural change to its appointment system. In addition, as the study involved a non-randomised QI project, it was not possible to take account of practice selection bias or other important contextual factors that may independently affect DNA rates.

### Comparison with existing literature

Most published literature on primary care DNAs focuses on the behaviour of the service user to explain non-attendance. Explanatory characteristics include young age and social deprivation,^8^^,^^15^^–^^17^ psychosocial problems,^16^ and less good markers of chronic disease control.^18^ Similarly, published interventions to reduce DNAs concentrate on behaviour change; these include getting patients to record their appointment times, reinforcing positive attending behaviours,^10^ and targeting service users at high risk of not attending appointments, also known as ‘hot-spotters’.^16^^,^^18^ Studies have explored appointment reminder systems such as SMS,^7^^,^^18^^–^^23^ or compared SMS and telephone reminders.^22^ In general, such interventions have only a modest impact, and generate associated financial and resource costs.

Other interventions, such as dynamic scheduling, attempt to increase efficiency by overbooking, based on predictions of DNA numbers;^24^ Dynamic scheduling suggests innovative practice but, in reality, most GPs operate a process of overbooking on a daily basis. System change — in particular, the reduction of advance booking for appointments — although only undertaken by one practice, had the largest sustained effect; this concurs with the literature around advanced-access models.^25^^,^^26^

### Implications for practice

This study demonstrates the impact of sharing practice organisational data in an easily accessible format, alongside QI training and coaching to support changes to GP appointment systems in one CCG.

Most practices chose to test behavioural interventions to reduce DNAs, despite being given information showing that booking delay is the major driver of DNA rates; this suggests that, although system change is more impactful, it requires major changes to work routines and is more challenging for providers. The one practice that changed its appointment system was forced to reassess access in response to a staffing crisis. It may take a crisis to justify taking the risks (real and perceived) that accompany changing ingrained working practices, for example, the COVID-19 pandemic has accelerated change in general practice, with a rapid adjustment to telephone triage for all appointments and remote consultations.

This study highlights some of the challenges of undertaking improvement work with independent organisations. Although it seemed clear to facilitators which interventions would be effective, each practice was encouraged to choose the components of their improvement work; this creates obstacles to maximising the impact and effective evaluation of improvement work. As Dixon-Woods has reported: *‘Having hundreds of organisations all trying to do their own thing also means much waste, and the absence of harmonisation across basic processes introduces inefficiencies and risks.’* ^27^

Much is already known about demand, capacity, and patient flow in primary care, hence the continuing wide variation in appointment systems is noteworthy — given that a major component of general practice business is the timely provision of GP appointments. Access remains a continuing challenge in primary care and the inconvenient truth is that the existing General Medical Services capitation-based contract provides little financial incentive to improve it. The unsatisfactorily high DNA rates in GP appointment systems illustrates the improvement mantra that *‘every system is perfectly designed to get the results it gets’*.^28^

DNAs should be viewed as an inevitable outcome of an appointment system, rather than a patient problem; to meaningfully reduce non-attendance, it is the appointment system itself that needs to be altered, not just the behaviour of its users.

## References

[1] Ellis DA, McQueenie R, McConnachie A (2017). Demographic and practice factors predicting repeated non-attendance in primary care: a national retrospective cohort analysis. Lancet Public Health.

[2] NHS (2019). Missed GP appointments costing NHS millions.

[3] Gregory A (2020). GP appointments missed by 20 000 patients each day. Failure to attend adds to queues and wastes £200m a year. The Times.

[4] British Medical Association (2020). Pressures in general practice.

[5] Palmer KT (2001). Practice matters. Notes for the MRCGP.

[6] Kaplan-Lewis E, Percac-Lima S (2013). No-show to primary care appointments: why patients do not come. J Prim Care Community Health.

[7] Martin C, Perfect T, Mantle G (2005). Non-attendance in primary care: the views of patients and practices on its causes, impact and solutions. Fam Pract.

[8] Sharp DJ, Hamilton W (2001). Non-attendance at general practices and outpatient clinics. BMJ.

[9] Healthwatch Tower Hamlets (2019). The Tower Hamlets GP report: satisfaction with booking appointments and waiting lists in GP Surgeries across the borough.

[10] Martin SJ, Bassi S, Dunbar-Rees R (2012). Commitments, norms and custard creams — a social influence approach to reducing did not attends (DNAs). J R Soc Med.

[11] Office for National Statistics (2012). 2011 Census: key statistics for local authorities in England and Wales.

[12] Department for Communities and Local Government English indices of deprivation 2015.

[13] Institute for Excellence in Health and Social Systems Knowledge Center/Worksheets. http://www.clinicalmicrosystem.org/knowledge-center/worksheets/.

[14] Lopez Bernal J, Cummins S, Gasparrini A (2017). Interrupted time series regression for the evaluation of public health interventions: a tutorial. Int J Epidemiol.

[15] Neal RD, Lawlor DA, Allgar V (2001). Missed appointments in general practice: retrospective data analysis from four practices. Br J Gen Pract.

[16] Shah SJ, Cronin P, Hong CS (2016). Targeted reminder phone calls to patients at high risk of no-show for primary care appointment: a randomized trial. J Gen Intern Med.

[17] Perron NJ, Dao MD, Kossovsky MP (2010). Reduction of missed appointments at an urban primary care clinic: a randomised controlled study. BMC Fam Pract.

[18] Steiner JF, Shainline MR, Bishop MC, Xu S (2016). Reducing missed primary care appointments in a learning health system: two randomized trials and validation of a predictive model. Med Care.

[19] Hallsworth M, Berry D, Sanders M (2015). Stating appointment costs in SMS reminders reduces missed hospital appointments: findings from two randomised controlled trials. PLoS One.

[20] Joseph C, Melder A (2018). Short message service (SMS) appointment reminders: a rapid review.

[21] Gurol-Urganci I, de Jongh T, Vodopivec-Jamsek V (2013). Mobile phone messaging reminders for attendance at healthcare appointments.. Cochrane Database Syst Rev.

[22] Perron NJ, Dao MD, Camparini Righini N (2013). Text-messaging versus telephone reminders to reduce missed appointments in an academic primary care clinic: a randomized controlled trial. BMC Health Serv Res.

[23] Fairhurst K, Sheikh A (2008). Texting appointment reminders to repeated non-attenders in primary care: randomised controlled study. Qual Saf Health Care.

[24] Creps J, Lotfi V (2017). A dynamic approach for outpatient scheduling. J Med Econ.

[25] Murray M, Berwick DM (2003). Advanced access: reducing waiting and delays in primary care. JAMA.

[26] Bostock N (2018). Next-day GP appointments three times as likely to be missed as same-day bookings. GP Online.

[27] Dixon-Woods M (2019). How to improve healthcare improvement — an essay by Mary Dixon-Woods. BMJ.

[28] Conway E, Batalden P (2015). Like magic? (‘Every system is perfectly designed …’). http://www.ihi.org/communities/blogs/origin-of-every-system-is-perfectly-designed-quote.

